# Supplementary material for: Endogenous protein tagging in medaka using a simplified CRISPR/Cas9 knock-in approach
Source: eLife. 2021 Dec 6;10:e75050. doi: 10.7554/eLife.75050 (PMC8691840; doi:10.7554/eLife.75050)
Supplement: Supplementary file 1. [file elife-75050-supp1.docx]

**File S1**

**Detailed protocol for cloning-free CRISPR/Cas9 knock-in of fluorescent reporters in Medaka**

Here, we provide a step-by-step protocol to tag endogenous genes with Fluorescent Proteins (FP) in medaka embryos (Iwamatsu, 2004, Naruse et al., 2004, Kasahara et al., 2007) using the CRISPR/Cas9 system (Jinek et al., 2012). The protocol consists of Cas9 mRNA guided endonuclease from the bacteria *Streptococcus pyogenes*, a single guide RNA (sgRNA) targeting Cas9 to the desired *locus*, and a repair donor containing the FP sequence of choice generated by PCR amplification. The Cas9 protein specificity comes from the choice of sgRNA, and the success of the FP insertion depends on the design of the donor in conjunction with the sgRNA used.

**PROTOCOL OVERVIEW**

-Cas9 mRNA *in vitro* transcription

-Design of sgRNA

-Design of PCR repair donor

-Synthesis of PCR repair donor

-Injection of the CRISPR/Cas9 mix in medaka embryos

-Medaka embryos screening and strain establishment

-Genotyping

-Positive control

-References

**A. CRISPR/Cas9 KI cloning-free reagents**

To avoid RNAse contamination, use clean solutions and materials. Use RNAse free H2O (no DEPC treated, as ThermoFisher #AM9938).

Cas9 mRNA *in vitro* transcription

While Cas9 with and without mSA can be used to generate KIs with high efficiency(Table S7), we have generated and validated the majority of KI lines presented in this manuscript using Cas9-mSA (all other Cas9 mRNA coding plasmids can be found in Table S4).

pCS2+ Cas9-mSA plasmid was a gift from Janet Rossant to Addgene (plasmid #103882) (Gu et al., 2018). Linearize 6-8µg of Cas9-mSA plasmid by Not1 HF restriction enzyme (NEB #R3189L). Run a 1.5% agarose gel and cut out the 8.8kb linearized fragment. Extract DNA using QIAquick Gel Extraction Kit (Qiagen #28115). Perform the *in vitro* transcription reaction using mMACHINE SP6 Transcription Kit (Invitrogen #AM1340).

Linearized DNA: approx. 100 ng/µl

2X NTP/CAP mix: 10 µl

10X SP6 Reaction Buffer: 2 µl

10X SP6 Enzyme: 2 µl

Incubate for 3-4h at 37°C

Add TurboDNase for 15 minutes at 37°C

Proceed directly to RNA clean up using RNAeasy Mini Kit (Qiagen #74104)

Elute in 35 µl RNAse free water

Measure concentration on a NanoDrop: expected concentration range 300-500 ng/µl

Design of sgRNAs

Single-guide RNAs (sgRNA) consist of the fusion of the two RNA molecules found in the CRISPR system: a *locus* specific crRNA and invariant tracrRNA to link the crRNA and the Cas9 protein. The sgRNA targets a sequence of 20nt (spacer sequence) upstream a PAM (Proto-Spacer Adjacent motif) which most commonly is an NGG sequence (Jinek et al., 2012).

-Find your gene of interest in a genome browser (as *Ensembl!*) and extract its sequence (including exons, introns, and flanking genomic regions). For medaka, the standard southern population genome is HdrR and this is compatible with the Cab strains maintained in the laboratory. Please note that a single polymorphism in the sequence recognized by the sgRNA can impair Cas9 cut if located near the PAM, but few variations in the homology arms should not impair Homology Directed Repair (HDR).

-Choose the site of insertion of the fluorescent reporter (usually N-terminus or C-terminus fusion). Some fusion can be non-functional (for example, in mammalian cells the Actb protein tagged in Ct with eGFP is nonfunctional) (Paix et al., 2019), therefore exploring the literature beforehand can be useful. If no information is available, we recommend trying both Ct and Nt fusions.

-Find a suitable spacer sequence (20nt upstream an NGG, look at both DNA strands). This can be done manually or by utilizing freely available tools (we recommend using CCTop at https://cctop.cos.uni-heidelberg.de:8043/ or CHOPCHOP at https://chopchop.cbu.uib.no/) (Labun et al., 2019, Stemmer et al., 2015). It is recommended to choose a spacer sequence as close as possible to the intended site of FP insertion. We recommend <10bp between the Cas9 cut (-3bp from PAM) and the site of insertion since it has been shown in mammalian cells and *C. elegans* that the insertion efficiency drops proportionally to the distance between the DSB and the site of insertion (Paix et al., 2017, Paix et al., 2019). In addition, inserting an FP away from the Cas9 cut increases the complexity of the PCR donor synthesis due to the necessity of using longer PCR primers. It is possible to insert an FP not at the extremities of the corresponding fusion protein, if it doesn’t impair any functional domains (use SMART website to find protein domain at http://smart.embl-heidelberg.de/). For secreted protein and Nt tagging, insert the FP downstream the signal peptide.

-Since it had been reported that a Guanine nucleotide just upstream the PAM increases sgRNA efficiency, whereas a Cytosine decreases the efficiency, it is recommended, when possible, to choose a sgRNA with a G upstream of the PAM (Doench et al., 2016). Also, when possible, choose a sgRNA with a GC content between 50-70% (Gagnon et al., 2014).

-Finally, verify that the selected sgRNA is specific for the desired *locus* by screening the list of off-target candidates. Several tools can be used (such as CCTop and CHOPCHOP). Generally, two or more mismatches in the 10bp upstream the PAM are sufficient to impair the Cas9 cut (Doench et al., 2016).

-Order the sgRNA from a commercial provider (for *e.g* Sigma-Aldrich: spyCas9 sgRNA, 3nmole, HPLC purification, no modification): Upon receipt, short spin the tubes and reconstitute to 100uM with H2O (30 µl for 3 nmol, ~3.2 µg/µl), mix by pipetting and store at -80C. Prepare a working dilution of 150-200 ng/µl in H2O and measure concentration on a Nanodrop, store at -80C.

Design of PCR donors

Following the site-specific DSB mediated by the sgRNA/Cas9, a repair donor (PCR product, see below) can be used to insert an FP sequence, in-frame with the protein coded by the targeted gene. Repair donors are generated via PCR (linear dsDNA) and contain the FP sequence, flanked by short (30-40nt) 5’ and 3’ homology arms, corresponding to sequences flanking the DSB and the site of FP insertion. PCR primers contain a biotin moiety on their 5’ ends, the homology arm sequence (30-40nt), followed by the FP sequences (19-21nt) for annealing with the template containing the respective FP coding sequence (excluding start and stop codon).

It is important to introduce silent mutations to prevent a subsequent Cas9 cut of the PCR donor and edited *locus* if the FP insertion does not impair the PAM or sequence recognized by the sgRNA (Doench et al., 2016, Paix et al., 2019, Paix et al., 2017). We recommend checking that the insertion will be in frame and does not create unintended codon changes by translating *in silico* the predicted gene fusion (as Expasy at https://web.expasy.org/translate/)

-Order the PCR primer with a commercial provider (for *e.g.* Sigma-Aldrich: 25nmole scale, desalted, 5’Biotin. No other modifications such as phosphorothioate bonds were added. 5’end Biotin is added to the primers to prevent *in vivo* concatemerization of the repair donor (and possible degradation as well) (Gutierrez-Triana et al., 2018). In addition, the Cas9-mSA has high affinity to the Biotin tags on the PCR donor and may help to bring the donor to the site of Cas9 cut (Gu et al., 2018). Upon receipt, reconstitute the oligo at 100uM in H2O, and store at -20°C.

Synthesis of PCR repair donors

PCR donors can be simply synthesized using a high-fidelity DNA polymerase (as NEB Master Mix Phusion with HF buffer #M0531L or NEB Master Mix Q5 #M0492L), a template plasmid containing the FP of interest (Table S4), and the custom designed primers containing the homology arms. We found that in more of >80% of the time, a PCR annealing temperature of 61.5°C provides a clean PCR product in the case of Phusion polymerase, 45s of elongation is enough to amplify the FP and homology arms (usually <800bp total), and 30 cycles are enough to get a sufficient concentration of repair donors.

-Mix in PCR tubes (thin walled): 0.1ul of plasmid template at 50-100 ng/µl (from a standard plasmid miniprep) + 0.25 µl of each PCR primers at 100 µM + 25 µl of 2X Phusion or Q5 Master Mix + 24.4 µl of H2O. Vortex, quick spin down and run the PCR as follow: 2 minutes at 98°C / 30s at 98°C, 30s at 61.5°C, 45s at 72°C, for 30 cycles / 10 minutes at 72°C / Hold at 10°C.

-Add a gel loading dye (as Bioworld Orange-G #10570024-1) to the finished PCR reaction and run on an agarose gel (1-1.5%) to check that a single band at the expected size is obtained.

-If the PCR product is not clean, we advise to run a PCR gradient to find the best annealing temperature. Make a PCR Master Mix with 0.8 µl of plasmid template at 50-100 ng/µl (from a standard plasmid miniprep) + 2 µl of each PCR primers at 100 µM + 200 µl of 2X Phusion or Q5 Master Mix + 195.2 µl of H2O, and split in 8*50 µl in PCR tubes. Run the PCR gradient as follow: 2 minutes at 98°C / 30s at 98°C, 30s with a gradient from 60°C to 72°C, 45s at 72°C, for 30 cycles / 10 minutes at 72°C / Hold at 10°C. Run an agarose gel and identify the annealing temperatures giving a clean product and pool them.

-Purified the PCR products with MinElute PCR Purification Kit (Qiagen #28004).) following the manufacturer protocol. Elute in 10 µl of H2O. Please note that we do not perform gel purification of the PCR products since PCR primers <70nt are removed with MinElute PCR Purification Kit.

-Check the concentration on a Nanodrop (approx. 50-100 ng/µl for the purification of a 50 µl PCR amplification) and store at -20°C.

**B. CRISPR/Cas9 insertion of fluorescent reporter**

Injection of the CRISPR/Cas9 mix in medaka embryos

The injection mix in medaka contains the sgRNA (15-20 ng/µl) + Cas9-mSA mRNA (150 ng/µl) + repair donor template (8-10 ng/µl). Male and female adult medakas are put together at the desired time and fertilized eggs are collected. The mix is injected in one-cell staged embryos (Iwamatsu, 2004). The embryos are raised at 28°C in 1X ERM (Embryo Rearing Medium) (Rembold et al., 2006, Seleit et al., 2017a, Seleit et al., 2017b).

Medaka embryos screening and strain establishment

Injected embryos are screened daily under a fluorescence stereoscope for signs of FP signal. Positive embryos are selected and grown separately. At hatch the fishes are screened once more, the brightest and most ubiquitous expressing fish are then grown to adulthood. Once adults the potential founders are outcrossed to the wildtype CAB strain. Embryos are collected daily and founder adults are designated.

Genotyping

Positive F1s for FP expression can be genotyped by PCR. Use primers annealing at ~250bp from each side of the site of FP insertion, in order to get a band of ~500bp for the wildtype allele and ~1200bp for insertion. This ratio allows the amplification of the insertion allele even with the competition of the wildtype allele. PCR bands corresponding to the insertion can be gel purified and sequenced, or directly purified and sequenced using primers annealing in the FP insert.

**C. Positive control**

We recommend for first users to tag the *myosinhc* gene (File S2) with mNeonGreen. Insertion efficiency is expected to be good (~20% of mosaic expression in surviving injected F0 embryos) and it is easy to screen due to the brightness of the fusion protein (Figures 1/2/S4). Use the following reagents:

-sgRNA AP253 (sequence 20nt upstream PAM): CATCTCTGCGTCAGTGCTCA

-PCR primers for donor: AP254 (5'Biotin-CCCCTCATTTTTAGACATCTCAAGTGCTACCATGGTCTCCAAAGGGGAGGAAG) and AP255 (5'Biotin-GCAGGGCCATAGGCCTCCATCTCTGCGTCAGTGCTCGAGCTTGATCCACTTGGAC).

-PCR reactions for Repair donor: use pAP27 as plasmid template. AP254/AP255 primers work well at an annealing temperature of 61.5°C for Phusion polymerase.

**D. References**

DOENCH, J. G., FUSI, N., SULLENDER, M., HEGDE, M., VAIMBERG, E. W., DONOVAN, K. F., SMITH, I., TOTHOVA, Z., WILEN, C., ORCHARD, R., VIRGIN, H. W., LISTGARTEN, J. & ROOT, D. E. 2016. Optimized sgRNA design to maximize activity and minimize off-target effects of CRISPR-Cas9. *Nat Biotechnol,* 34**,** 184-191.

GAGNON, J. A., VALEN, E., THYME, S. B., HUANG, P., AKHMETOVA, L., PAULI, A., MONTAGUE, T. G., ZIMMERMAN, S., RICHTER, C. & SCHIER, A. F. 2014. Efficient mutagenesis by Cas9 protein-mediated oligonucleotide insertion and large-scale assessment of single-guide RNAs. *PLoS One,* 9**,** e98186.

GU, B., POSFAI, E. & ROSSANT, J. 2018. Efficient generation of targeted large insertions by microinjection into two-cell-stage mouse embryos. *Nat Biotechnol,* 36**,** 632-637.

GUTIERREZ-TRIANA, J. A., TAVHELIDSE, T., THUMBERGER, T., THOMAS, I., WITTBRODT, B., KELLNER, T., ANLAS, K., TSINGOS, E. & WITTBRODT, J. 2018. Efficient single-copy HDR by 5' modified long dsDNA donors. *Elife,* 7.

IWAMATSU, T. 2004. Stages of normal development in the medaka Oryzias latipes. *Mech Dev,* 121**,** 605-18.

JINEK, M., CHYLINSKI, K., FONFARA, I., HAUER, M., DOUDNA, J. A. & CHARPENTIER, E. 2012. A programmable dual-RNA-guided DNA endonuclease in adaptive bacterial immunity. *Science,* 337**,** 816-21.

KASAHARA, M., NARUSE, K., SASAKI, S., NAKATANI, Y., QU, W., AHSAN, B., YAMADA, T., NAGAYASU, Y., DOI, K., KASAI, Y., JINDO, T., KOBAYASHI, D., SHIMADA, A., TOYODA, A., KUROKI, Y., FUJIYAMA, A., SASAKI, T., SHIMIZU, A., ASAKAWA, S., SHIMIZU, N., HASHIMOTO, S., YANG, J., LEE, Y., MATSUSHIMA, K., SUGANO, S., SAKAIZUMI, M., NARITA, T., OHISHI, K., HAGA, S., OHTA, F., NOMOTO, H., NOGATA, K., MORISHITA, T., ENDO, T., SHIN, I. T., TAKEDA, H., MORISHITA, S. & KOHARA, Y. 2007. The medaka draft genome and insights into vertebrate genome evolution. *Nature,* 447**,** 714-9.

LABUN, K., MONTAGUE, T. G., KRAUSE, M., TORRES CLEUREN, Y. N., TJELDNES, H. & VALEN, E. 2019. CHOPCHOP v3: expanding the CRISPR web toolbox beyond genome editing. *Nucleic Acids Res,* 47**,** W171-W174.

NARUSE, K., HORI, H., SHIMIZU, N., KOHARA, Y. & TAKEDA, H. 2004. Medaka genomics: a bridge between mutant phenotype and gene function. *Mech Dev,* 121**,** 619-28.

PAIX, A., FOLKMANN, A. & SEYDOUX, G. 2017. Precision genome editing using CRISPR-Cas9 and linear repair templates in C. elegans. *Methods,* 121-122**,** 86-93.

PAIX, A., RASOLOSON, D., FOLKMANN, A. & SEYDOUX, G. 2019. Rapid Tagging of Human Proteins with Fluorescent Reporters by Genome Engineering using Double-Stranded DNA Donors. *Curr Protoc Mol Biol,* 129**,** e102.

REMBOLD, M., LAHIRI, K., FOULKES, N. S. & WITTBRODT, J. 2006. Transgenesis in fish: efficient selection of transgenic fish by co-injection with a fluorescent reporter construct. *Nat Protoc,* 1**,** 1133-9.

SELEIT, A., KRAMER, I., AMBROSIO, E., DROSS, N., ENGEL, U. & CENTANIN, L. 2017a. Sequential organogenesis sets two parallel sensory lines in medaka. *Development,* 144**,** 687-697.

SELEIT, A., KRAMER, I., RIEBESEHL, B. F., AMBROSIO, E. M., STOLPER, J. S., LISCHIK, C. Q., DROSS, N. & CENTANIN, L. 2017b. Neural stem cells induce the formation of their physical niche during organogenesis. *Elife,* 6.

STEMMER, M., THUMBERGER, T., DEL SOL KEYER, M., WITTBRODT, J. & MATEO, J. L. 2015. CCTop: An Intuitive, Flexible and Reliable CRISPR/Cas9 Target Prediction Tool. *PLoS One,* 10**,** e0124633.
