# Supplementary material for: Endogenous protein tagging in medaka using a simplified CRISPR/Cas9 knock-in approach
Source: eLife. 2021 Dec 6;10:e75050. doi: 10.7554/eLife.75050 (PMC8691840; doi:10.7554/eLife.75050)
Supplement: Supplementary file 2. [file elife-75050-supp2.docx]

**File S2**

**Detailed sequence design for *mNeonGreen-HAtag-Linker-myosinhc* tagging**

>*myosinhc* (Transcript: ENSORLT00000002485.2): coding exons are in grey, PAM in bold, sequence recognized by the sgRNA is underlined.

CAAACATGCTTAGTAGGCAATATAAGCACTGTAAATATCAATGTCACAGGATTAGAGACAACATGACTAGAATACATTACCAAATTGAGCAGGGCACACCTACCACACAGTCAGTCCTCCCTCTATAAAAGAGAGCACTTAATGCTCTGGGAATAACTGGTGGGCTTCCAACTCAGGTGAGTGCAAAAATGTATTAAGCCTTATTTTGCAAATTTCTGTTGTTCAAATGCTATTTGGAATATAATTGTTCCCAAAGGAAATCTATAAACAAATGGGATGCATTTAGATTTGTTTTAAGTCATTGTATTGTATTAAGATGCTTGTGCTTTACAATTTAATAGTGGAAACAACATAACACCGTAAGATAATCTGCTGAGAGCCAAACAGGTAGACATTTTTTTTCCAAATTCTTTACCATGTTTCTTGTTTTGAGCAAGTGAACAAAAAATAAATAAATCAATGATTGTCACCCCTCATTTTTAGACATCTCAAGTGCTA**CCA**TGAGCACTGACGCAGAGATGGAGGCCTATGGCCCTGCGGCCATCTACCTCCGGAAACCAGAGAAGGAGAGGATTGAGGCTCAGGCAGCTCCTTTTGATGCCAAAACGGCCTACTTTGTGGCAGAGCCAGAGGAGATGTATCTGAAGGGAAAACTTATCAAAAGGGAGGGTGGCAAAGCCACTGTTGAGACAGTAACAGGAAAGGTTGGTAAACTAAGGCTTTTGTTTACTCTTACATTTCACTTACCAAGTAAAATGTAATCAACTCTATGTCATTTAAGTAAACCATAAATCATGTGAAACACTGGTTAAAAATGTTCTTATTTATTCTAACTCTTGTTTAGACTTTAACTGTGAAAGAGGAGGACATCCATCCAATGAACCCTCCCAAGTTTGATAAAATTGAGGACATGGCCATGATGACCCACCTTAATGAACCCGCTGTGCTGTACAACCTCAAAGAACGTTTTGCATCATGGATGATCTATGTATGTAAATGACTTAGACAAGCACAGAGAAAAAATGTTTTATTTTATATTGGTGTAACGTAACGTTTCAAAAGTTTACTATTTGGTTTAGCAATAACTCTACTGTCTTTACAGACTTACTCTGGGCTGTTTTGTGTTGTCGTGAACCCATACAAGTGGCTTCCGGTGTACGATGCTCAGGTTGTCAATGCCTACAGAGGCAAGAAGAGAATTGAGGCTCCCCCTCATATCTTTTCCATCTCTGACAATGCCTATCAGTTCATGCTCACTGGTAAGATAGTTGCACAACAGAGAAAGCAGCACACACAAATAAAACTCCACAGGTGTGGTTCACTGTGCGCATGCTTGCATTTCAGATCGTGAGAATCAGTCTATCCTTATCACGTGAGTATAAGGCAACATGCCAAGAGTCCCTATGAAATTTTAGGATTAATGTGCCACATAAAGGCTGTGTCTTACATTCTCAGTGGAGAATCTGGTGCAGGAAAGACTGTCAACACCAAGCGTGTCATCCAGTACTTTGCAACAATTGCAGTGGCTGGAGGAAAGAAGTCTGAGGGAAGCTCAGGAAAAATGCAGGTACAGAAGAATGCAAAACTTCTCACAATAGTAGGTTGTTGTTTAGTTAAAACACATTAGTTTGTTCTTTCTGCAGGGTTCACTGGAAGATCAAATCATTGCAGCCAACCCTCTGCTGGAGGCCTATGGTAATGCCAAGACTGTGAGGAATGATAACTCTTCTCGCTTTGTGAGTTTAAAAAAAGTCCTCAAATATTGTTAATGTTCTTATTACTTAGAATTTGTCTGACATTGTCTTGAAAATTTTCAGGGTAAATTTATCAGAATCCACTTTGGTTCTACTGGTAAACTTGCTTCAGCTGATATTGAAACATGTAAGTTAGACAGCATTTCTATAATTGTAACTCCTTGGCCTGCATCGGTGCAGGACTAACATTAACAACATGCAATAAATTTCAGATCTGCTGGAGAAGTCTCGTGTCACCTTCCAGTTGTCTGCTGAGAGGAGCTACCATATCTTTTACCAGCTGATGACGGGCCACAAACCTGAGCTCCTTGGTATGATACAAGACAAATAATTACACTTAGGATGGAATAGGTAAAAAAAATCCTCTTTACTTGTGTTATTTTCAAGGTACATAAAGAGAAAACTACTTTTCCTTCTAGAGCAAATCTGTTACCAAAAATGTACATGCAAAAGAGAATGTTCCCATTTCTGATATTCCATTTTTTCCCCTTTAGAGGCTCTTCTTATCACCACCAACCCGTATGATTATCCAATGATCAGCCAGGGTGAAATCACTGTCAAGAGCATCAATGACGTTGAGGAGTTCATTGCAACTGATGTAAAAGAAATACTTAATACTTGTAAATTATAATTTGCAAAATTGTAATCATTAATAATTGAACTATTTTAATTTTTAGGTCTGTACATATCAATTTATGTTATGGGATTTTTTAAAACAATTGTCTCTGTATGTTTGCAGACTGCCATTGACATATTGGGATTCAGTGCTGAGGAAAAAATGGGCATCTACAAGCTAACTGGTGCTGTGATGCATCATGGTAACATGAAGTTTAAGCAGAAGCAGCGAGAGGAGCAGGCAGAACCTGACGGCACAGAGGGTAAATTAACTGTTAATAATGATAGATCAATAATGACTTAGTCTCAGTTTGTTTTACATTTTATAAGATAAGATAAGATAAGATAAAGATACTTTATTGATCCCAGCTTGGGAAATTCAGTTGTTGCAGCGTCGGCATTATAGGCTCAAGATACAAACATACACTCAATAGACGACAACAGAGAACCAGAACAATCAATCGGAAAGGAACATCAGTGAATTAGAACATTTGCATTGCGTGCCACATCAGGAGAGAAAAACAGACAGACCATAAATGACTTTATCTAGTGCCCATGGATGACAAGTTATAGAGTCCAATGGAGTGAGGTATGAAAGATCTCCTGAAGCGGTTTGTGTGCCAGCGAAGCTGCCTTAGCCTGTTGGAGAAGGAGCTCCTCTGACCATCCAGTACATCCAGTACAGTTTTACTCAACACAGAGGAAAATGCTTTGTTAAATCCAGAATTGTTTTTTCCATCAGTGGCTGATAAAATCGCCTACCTGATGGGCCTAAACTCAGCTGACATGCTGAAAGCTCTGTGCTACCCAAGAGTCAAGGTTGGAAATGAGATGGTCACCAAAGGTCAGACTGTCCCACAGGTGACTCACTCAAACAGTTTATGGGGATTGTTTACTTAAAAGAAATATTGATTTGAAATAATTTCATTAAAGTGCTAAACTTTTTTTTAGGTTAACAATGCTGTCAGTGCTCTGTGCAAGTCTGTCTATGAGAAAATGTTCTTGTGGATGGTCATCCGCATCAATGAGATGTTGGACACAAAGCAGCCAAGACAGTTCTTCATTGGAGTGCTGGACATTGCTGGATTTGAAATCTTTGATGTGAGTTGCAGTGGGTAACAAATTAGGCTATCAGTCATTTTCCTATATTTTAAGGAGTTAAACCTGTTTTGTTTTCAACCAAATAATCCTTTTTTTTAACCTGCAGTACAACAGCTTGGAGCAGCTCTGCATCAACTTCACCAATGAGAAACTGCAACAGTTTTTCAATCACCACATGTTTGTCCTGGAGCAAGAGGAGTACAAGAAAGAAGGCATTGAATGGGAGTTCATTGACTTTGGCATGGACTTGGCTGCCTGCATTGAGCTTATTGAGAAGGTATACATTCTGTTCAGAAATGTTCCCGTTGTGAGATTAGTAACGTTCTACCTATTTATCCTTGTGTAATTACTTTTTATGATTAGGAAAAAAAAGTTTTTTCATCTTGGTCTGCTATTTTAATGCCAAATTATACTTTTTCCAAGCCAATGGGCATCTTCTCCATCCTTGAAGAGGAGTGCATGTTCCCCAAGGCTACAGACACCTCATTTAAGAACAAACTGCATGATCAGCATCTTGGCAAGACCAAGGCCTTTGAGAAGCCTAAACCTGCAAAAGGAAAAGCTGAAGCTCACTTTGCCCTGGTTCATTATGCTGGAACAGTGGATTACAACATAACTGGCTGGCTGGACAAAAACAAGGACCCACTGAACGACTCAGTGGTGCAGCTCTACCAGAAGTCTTCAAACAAACTGCTGTGCTACCTCTATGCAGCCCATGCAGGAGCTGAAGGTAAGACTTTAACAAAAAGCTGTTAAATGATAGTTTGTTTATATCTAGATAAATGCACATCATTAAAATTCATGTGTAGTATGACTAATTGTGTTATATATATCAAAAACAAAACAAAATTAGAAGCCTCTGGTGGTGCCAAAAAGGGTGGTGGAAAGAAGAAGGGTGGTTCCTTCCAAACTGTGTCAGCACTTTTCAGGGTAAGTTTATTTATTAAGACATATTTAAGATCTGTAGATTTTATTTTTTTACAACTGGCACTGAAAAAAATTATGTGCTTCATTCATCAGGAAAATCTTGGCAAGCTGATGACCAATTTGAGAAGCACTCATCCACATTTTGTCCGCTGCTTGATTCCAAATGAATCTAAAACCCCAGGTCTGTTGTGGTACCTTATAACTACGTCAAAGTCAATATATGATTTGAAAAATTCCCAGAAACAATAAAAAGGGGCTACCCTTTCTTTTACAATTCCTTGTATTTTAAAACTTCATCCATTCTGAATGATTAGGTCTTATGGAGAACTTCTTGGTCATCCACCAGCTGAGGTGTAATGGTGTGCTGGAGGGCATCAGAATCTGCAGAAAGGGTTTCCCCAGCAGAATCCTCTATGGTGACTTCAAGCAGAGGTAAATGGCAGATTTACTGGATGTAATTTAAAAAAAAAATATATATATATATATATATGTGTGTGTGTATATATATAAATATATATATATATATATATATATATATATATATATATATATATATATATATATATATATATGTGTGTGTGTGTGTATATATATATAAATATATAACTTTGCTAAATAATATTAATTAGAAAGGAAAATGTGGATAAATCTACTTTTATCTTACAGATACAAAGTATTGAATGCCAGTGTCATCCCTGAGGGACAGTTCATCGACAACAAGAAAGCTTCAGAGAAGCTGCTTGGTTCCATTGATGTTGACCACAGTCAGTACAAGTTTGGACACACTAAGGTAGGTTTCTATAAAATAGTTATGTAAATTGGCTCAAGTCCCAGCTGGGGAACCTGAAACAGAACACCAATGTGGGACCCTGTGTGGAGATTGCATGTTCTTCCTGTCCATGTTTGGGTTTTCTCCTCCATTCAAAAACGTGCTTCATAGGTTAGCAGATTACTCTGAATTGTCCCTAGGTGTGAATGTGAGTGTGCTTGGGCCTATCCAGGCCACAAGTGACTGGGATAGGCTCCAGCAGCCCTGTGACCCCGAAAGGGACAAAATGGCTTAATGAATGAATGAATGAATGAATGAATGAATGAATGAATGAATGAATGAATGAATGAATGAATGAATGAATGAATGAATGAATGAATGAATGAATGAATGAATGAATTGGCTTTTGGACGAGGTGTAACCTATCATGTCAATATTTCAACACAGGTGTTCTTCAAAGCTGGTCTGCTGGGTACACTTGAGGAGATGAGAGATGAGAAGCTGGCTGAGCTGGTGACCATGACTCAGGCTCTCTGCAGAGGGTTCCTTATGCGGAAAGAGTTTGTTAAGATGATGGAAAGGAGGTAAGACTCCCACATATTAGCTTACACAGGAATCACTGTTCAAATTAAAAGGCTGTTCAGGAAGCATAATGTCAACTTATCTGTGTTGCTCTTTAGAGATGCAATCTTCACCATCCAGTACAACGTCCGTTCATTCATGAATGTAAAGAACTGGCCATGGCTTAAACTCTACTTCAAGATCAAGCCTCTTCTGAAGAGCGCTGAGACTGAAAAGGAGCTGCAGGAGATGAAAGGAAACTATGAGAAGATGAAAACAGACCTGGCTGCTGCTTTGGCCAAGAAGAAGGAACTGGAGGAGAAAATGGTTTCTCTGCTGCAGGAAAAGAATGACCTGCAGCTTCAAGTAGCTGCTGTAAGTAAAAAATATGAAGACATATCATGAACAAAGCTCATAAAATAATGTCTTATATCAATATTTAGGAATAAAAATAATGTGACAAAATATGTTTGATATTAGATGTCAGAAATGTAGAAATGTGTCTAAACTACAGGAAACAGAGAATCTCTCCGATGCTGAGGAAAGGTGTGAGGGACTCATTAAGAGTAAAATCCAGCTGGAGGCCAAACTCAAAGAGACCACTGAGAGACTGGAGGATGAAGAGGAAATCAATGCTGAGCTGACTGCTAAGAAGAGGAAGCTGGAGGATGAATGTTCTGAACTGAAGAAGGACATTGATGACTTGGAGCTCACCCTGGCTAAAGTGGAGAAGGAGAAACATGCCACTGAAAACAAGGTTAATGTCAAACTATTGTACACATTTTATATAAATTCAAATTATAAAATTTGACCTTTTATGGTACTCTAAGGTGAAAAACCTGACTGAGGAAATGGCTTCTCAAGATGAGTCCATTGCAAAGCTGACCAAGGAGAAGAAAGCCCTCCAAGAGGCCCATCAACAAACACTGGATGATCTCCAGGCAGAGGAAGACAAAGTCAACACTCTGACCAAAGCCAAGACCAAGCTGGAACAGCAAGTGGATGATGTAAGAAAAATTAATGTTTTGACAAATTTGACTGAAATAGATGATCTGAAGAAGTTATTTAACAATGAAATATTTTTATCTGACAGCTTGAAGGTTCATTGGAGCAAGAGAAGAAGATCCGCATGGATCTTGAGAGAGCCAAGAGGAAGCTGGAAGGAGATCTGAAACTAGCCCAGGAATCCATAATGGATCTGGAAAATGACAAGCAGCAGTCTGATGAGAAGATCAAGAAGTAAGAAAAAATGTGTTTTGATGCAAAATTAAAGGGTTTTTTGTGGTTTTAAAGTAAACGGGGACATTCCTTTTTGAAATCCAGAAAGGACTTTGAAATCAGTCAGCTCCTCAGCAAGATTGAAGATGAACAATCCCTTGGTGCTCAGCTTCAGAAAAAGATCAAGGAACTCCAGGTACAATTTTTGCAGCCATTATTGGCTAGATTATTGTGGGTTGTTGTAATACACTACTTATATATATTAAGAGTATTGTATATAATACTCTACTTATAATACAAAACTTTAAAAAATTTAATTTAGGCTCGTATTGAGGAGCTGGAAGAGGAAATTGAGGCTGAGAGAGCTGCCCGGGCCAAAGTAGAGAAGCAGAGAGCTGATCTCTCCAGGGAGCTTGAGGAGATCAGTGAGAGGCTTGAAGAAGCTGGAGGGGCAACAGCTGCTCAGATTGAGATGAACAAGAAGCGGGAAGCTGAGTTCCAGAAGCTGAGGAGAGATCTTGAGGAGTCCACACTGCAGCATGAAGCTACTGCAGCAGCTCTCCGCAAGAAGCAGGCCGACAGCGTTGCAGAGCTGGGAGAACAGATCGACAACCTGCAGCGTGTCAAGCAGAAGCTGGAGAAGGAGAAGAGCGAGTACAAGATGGAGATTGATGATCTCAGCAGCAACATGGAGGCAGTTGCTAAATCAAAAGTGAGAATTGTCATTGATTTTCTTTTGAGAACTCTGATAAATATAACCTTATAACGTTTGCTTTTATTTTTGTCTATTTCTTTTAAGGGTAACCTGGAGAAGATGTGCAGAACTCTTGAGGACCAGCTGAGTGAGCTCAAGGCCAAAAATGATGAAAATGTTCGCCAACTGAATGACATCAATGTACAAAAGGCAAGACTTCAAACGGAAAATGGTGAGTAACTTGAAACAAAAAGGTATTTTTTAAGATTACAATTAAAATTAAGTAAACTGTTTTTGCTTGTTATTATCAGGTGAATTTGCTCGCCAGCTTGAGGAAAAAGAAGCTCTAGTTTCTCAGCTGACCAGAGGCAAACAGGCCTTCACTCAGCAGATTGAGGAGCTGAAGAGACATGTGGAGGAGGAAGTGAAGGCATGAAAACCTTATGCATTCTATTAATCTCAAGCTTCTTGGAGGCTGTAAAAATTCTGGGCCCCCTAAATAAATTTTAATAAATCATTTTAAAACAATCAAGTCACTGAAGAGTTCTGTGATTTCTCCAGGCCAAGAACGCTCTGGCTCATGCTGTCCAGTCAGCCCGTCATGACTGTGATCTGCTCAGAGAGCAGTTTGAGGAGGAGCAGGAGGCCAAGGCTGAGCTGCAGAGAGGAATGTCCAAGGCCAACAGTGAGGTGGCTCAGTGGAGAACCAAATATGAAACTGATGCCATCCAGCGCACTGAGGAACTGGAGGAGTCCAAGTTAGTCAAAGCAGTTCATCATTTCATCAAACTATTCTGTCTCTCACTTCTTTTAAAACATTCACTCCATCCATTTGCTGCAGGAAAAAGCTTGCCCAGCGCCTGCAGGAGGCTGAGGAGTCCATTGAGGCTGTGAACTCCAAGTGTGCCTCTCTGGAGAAGACCAAGCAGAGGCTGCAGGGTGAAGTGGAGGACCTCATGATTGATGTGGAGAGAGCTAATGCTCTTGCTGCAAACCTGGACAAGAAACAGAGAAACTTTGACAAGGTACCATGTTCATCTGACAGTTAAAAAAAGTAAAACATGTTGGGAAGATACACTTACTTTAGTAACAAACCTGCAACCAATGGTTAACCTCTAGGTCCTGGCAGAATGGAAGCAGAAGTATGAGGAGAGCCAGGCAGAGCTTGAAGGAGCTCAAAAAGAGGCTCGCTCTCTCAGCACAGAACTGTTCAAGATGAAGAACTCCTACGAGGAGGCCCTGGATCAGCTGGAGACCATGAAGAGGGAGAACAAGAACCTGCAGCGTATGTCAACAATCTATATTGATAACAAAGAATTATTTATAACCAATTGGAAAGATACAAATTTTGCTAACTTTTCTTGAATTAACATGACAGAGGAGATCTCAGACTTGACTGAACAGATTGGTGAGAATGGAAAAAGCATCCATGAGTTGGAGAAAGCCAAAAAGACTGTTGAAAATGAAAAGATTGAACTTCAGACAGCTCTTGAGGAGGCAGAGGTAAAATTTTGTAAACAAGATATTGTGTATTAAGTTTACACTTCACAATTTTTTTCACAGTTGTCTTGTTAATTAACTTTGGCCTTTTCCTTCAGGGCACTCTAGAACATGAGGAATCCAAGATTCTTAGAGTACAGCTTGAGCTCAACCAGGTCAAAGGTGAGATTGACAGGAAGCTGGCAGAGAAGGACGAGGAGATGGAACAGATCAAGAGGAACAGCCAGAGGGTGATTGAATCCATGCAGAGCACTCTTGATGCTGAGGTCAGGAGCAGAAATGATGCCCTGAGAGTCAAGAAGAAGATGGAGGGAGACCTGAATGAGATGGAGATTCAGCTCAGCCATGCCAACAGGCAGGCTGCTGAGGCCCAGAAACAACTCAGGAATGTTCAGGGACAACTCAAGGTGAGATTTATCTTCAATTAAAGCTACTAACTTATGAACATCTTTACACAACTTTATGAAAAAAAAATTGTTTCAAAGTAGCAGACTTCTAAAAGAACTTCTAGAAAGTTTTATCAGCGTCACGCATGCTCTTATTGTTGTTGTCAGGATGCCCAACTGCACCTTGATGACGCTCTGAGAGGACAAGAAGACATGAAAGAACAGGTTGCCATGGTGGAGCGTAGGAATGGTCTGATGTTGGCTGAGATTGAGGAGTTGAGAGCCGCTCTGGAACAGACAGAGAGAGGACGTAAAGTGGCTGAACAGGAGCTGGTGGATGCCAGTGAACGTGTTGCATTGCTTCACTCCCAGGTTGATATTTAACAAAAACTGAAAAACTGTGCACAACTCAAGATTTGAGAAACGTTCACAGGCAATCAGGCAATAATTACATTTATTTTCAGAACACCAGTCTTCTGAACACCAAGAAGAAGCTGGAGGCTGACTTTGTTCAGGTTCAGGGTGAAGTGGATGATGCTGTTCAAGAAGCAAGAAATGCAGAGGAGAAAGCCAAGAAGGCCATTACTGATGTAAGTTTATTCTTGGTACATTTCAAACATTGGATGCTCGTGTTTACCACTGATTTCTTTTCAGTTTAAATTAAATCAGACACCTGTTATCCCTTTCAGGCTGCCATGATGGCTGAGGAGCTAAAGAAGGAGCAGGACACCAGCTCTCATCTGGAGAGGATGAAGAAGAACCTGGAGGTCACAGTGAAGGACCTGCAGCACCGTCTGGATGAAGCTGAGAACCTGGCTATGAAGGGTGGAAAGAAGCAGCTCCAGAAACTGGAGTCCAGGGTAGATACATAGTGTCCCTAATTTTGCAATATTTATATTGTGTGGGTTTGTTCTGTCTGTAGTGTACTAACTCTGCTCTTTAACATCCTATTATAGGTGCGTGAATTAGAGGCTGAAGTTGAAACTGAGCAAAGACGTGGAGCTGATGCGGTTAAAGGCGTTCGCAAATATGAAAGAAGAGTGAAGGAGCTCACATACCAGGCAATTCAAACTCTTTTTACAGATTTCCGTCTTAATTTTGAATAGATTTAATTTTAAGTCAAAACTTTTTAATCTGTGTAGACTGAGGAGGACAAGAAAAATGTAAGCAGACTCCAGGATCTGGTGGATAAGTTACAGCTCAAAGTAAAGGCCTATAAGAGACAGGCTGAAGAGGCTGTAAGTAACATTCTATTAGGATTTGGTTAGGAAAATAAAAATGTAACTAGTTTAACAACCACATTATTTCACTCTTTTCATTTTATAGGAGGAGCAGGCCAACACCCATATGGCCAGACTTAGAAAAGTCCAGCATGAACTTGAGGAAGCTCAGGAACGTGCCGACATCGCAGAGACCCAAGTCAACAAGCTGAGAGCCAAAAGTCGTGATATTGGAAAGGTAACTTTTCACTCTGTGATATTTTCATATATCTAATTAATGATAAGCACATGTGTAATTTATTACAGTTCAAATGTAAACAAAAAGAATTGTGTTACAAAGAATTCCCAAAATTATATTGCGAACAACAGGTTTCTCTCACAACCTCTTACCCAACAAAATGTCTTCTTCTTTCAGGGTACAGACTCAGCTGAGTAACAGTCAAGATCCCCTGGGACAGAGACAGATTCATATAATATGAACCCCCTCTGTTCATCTTTAGCTTATAAATAAATGAACTATACTTGAAACAAATTCCGTGTATTGTTCATTTACTTCTTTATTGTGGTCAGAGTCAATAATAGCTGAAGATGTCTTCAGGCAAGATGCTGTATATTCTTTGAGTGCTTGTCAGTCTATCACAAATCACCTCATCTCATAAAACTGTATGAGAGTTAATATAAGACAAAGATAAATAATCAGTCATATCATTGCTGTAATTCAAGTTGTAACATGTATTAATGCTCTTTCATTTGAGGCTGCAGCGTGGGGAGGCTGGTTACAAATTTAAATCAAACATTCAAACGAAATTAAGACCTTTTCTGTTTGGTATTTTGCTTTTATTATTATACATCTTCATTATCCTACCTTAACACGAGTCACAAGAGCACTGGGCTTACTTACACATGATGACAACAGTAAAACAGATAAACACAAAA

>Sequence targeted by sgRNA AP253 (20nt upstream PAM)

CATCTCTGCGTCAGTGCTCA

>*myosinhc* (Transcript: ENSORLT00000002485.2): CDS

ATGAGCACTGACGCAGAGATGGAGGCCTATGGCCCTGCGGCCATCTACCTCCGGAAACCAGAGAAGGAGAGGATTGAGGCTCAGGCAGCTCCTTTTGATGCCAAAACGGCCTACTTTGTGGCAGAGCCAGAGGAGATGTATCTGAAGGGAAAACTTATCAAAAGGGAGGGTGGCAAAGCCACTGTTGAGACAACTTTAACTGTGAAAGAGGAGGACATCCATCCAATGAACCCTCCCAAGTTTGATAAAATTGAGGACATGGCCATGATGACCCACCTTAATGAACCCGCTGTGCTGTACAACCTCAAAGAACGTTTTGCATCATGGATGATCTATACTTACTCTGGGCTGTTTTGTGTTGTCGTGAACCCATACAAGTGGCTTCCGGTGTACGATGCTCAGGTTGTCAATGCCTACAGAGGCAAGAAGAGAATTGAGGCTCCCCCTCATATCTTTTCCATCTCTGACAATGCCTATCAGTTCATGCTCACTGATCGTGAGAATCAGTCTATCCTTATCACTGGAGAATCTGGTGCAGGAAAGACTGTCAACACCAAGCGTGTCATCCAGTACTTTGCAACAATTGCAGTGGCTGGAGGAAAGAAGTCTGAGGGAAGCTCAGGAAAAATGCAGGGTTCACTGGAAGATCAAATCATTGCAGCCAACCCTCTGCTGGAGGCCTATGGTAATGCCAAGACTGTGAGGAATGATAACTCTTCTCGCTTTGGTAAATTTATCAGAATCCACTTTGGTTCTACTGGTAAACTTGCTTCAGCTGATATTGAAACATATCTGCTGGAGAAGTCTCGTGTCACCTTCCAGTTGTCTGCTGAGAGGAGCTACCATATCTTTTACCAGCTGATGACGGGCCACAAACCTGAGCTCCTTGAGGCTCTTCTTATCACCACCAACCCGTATGATTATCCAATGATCAGCCAGGGTGAAATCACTGTCAAGAGCATCAATGACGTTGAGGAGTTCATTGCAACTGATACTGCCATTGACATATTGGGATTCAGTGCTGAGGAAAAAATGGGCATCTACAAGCTAACTGGTGCTGTGATGCATCATGGTAACATGAAGTTTAAGCAGAAGCAGCGAGAGGAGCAGGCAGAACCTGACGGCACAGAGGTGGCTGATAAAATCGCCTACCTGATGGGCCTAAACTCAGCTGACATGCTGAAAGCTCTGTGCTACCCAAGAGTCAAGGTTGGAAATGAGATGGTCACCAAAGGTCAGACTGTCCCACAGGTTAACAATGCTGTCAGTGCTCTGTGCAAGTCTGTCTATGAGAAAATGTTCTTGTGGATGGTCATCCGCATCAATGAGATGTTGGACACAAAGCAGCCAAGACAGTTCTTCATTGGAGTGCTGGACATTGCTGGATTTGAAATCTTTGATTACAACAGCTTGGAGCAGCTCTGCATCAACTTCACCAATGAGAAACTGCAACAGTTTTTCAATCACCACATGTTTGTCCTGGAGCAAGAGGAGTACAAGAAAGAAGGCATTGAATGGGAGTTCATTGACTTTGGCATGGACTTGGCTGCCTGCATTGAGCTTATTGAGAAGCCAATGGGCATCTTCTCCATCCTTGAAGAGGAGTGCATGTTCCCCAAGGCTACAGACACCTCATTTAAGAACAAACTGCATGATCAGCATCTTGGCAAGACCAAGGCCTTTGAGAAGCCTAAACCTGCAAAAGGAAAAGCTGAAGCTCACTTTGCCCTGGTTCATTATGCTGGAACAGTGGATTACAACATAACTGGCTGGCTGGACAAAAACAAGGACCCACTGAACGACTCAGTGGTGCAGCTCTACCAGAAGTCTTCAAACAAACTGCTGTGCTACCTCTATGCAGCCCATGGTGGTGGAAAGAAGAAGGGTGGTTCCTTCCAAACTGTGTCAGCACTTTTCAGGGAAAATCTTGGCAAGCTGATGACCAATTTGAGAAGCACTCATCCACATTTTGTCCGCTGCTTGATTCCAAATGAATCTAAAACCCCAGGTCTTATGGAGAACTTCTTGGTCATCCACCAGCTGAGGTGTAATGGTGTGCTGGAGGGCATCAGAATCTGCAGAAAGGGTTTCCCCAGCAGAATCCTCTATGGTGACTTCAAGCAGAGATACAAAGTATTGAATGCCAGTGTCATCCCTGAGGGACAGTTCATCGACAACAAGAAAGCTTCAGAGAAGCTGCTTGGTTCCATTGATGTTGACCACAGTCAGTACAAGTTTGGACACACTAAGGTGTTCTTCAAAGCTGGTCTGCTGGGTACACTTGAGGAGATGAGAGATGAGAAGCTGGCTGAGCTGGTGACCATGACTCAGGCTCTCTGCAGAGGGTTCCTTATGCGGAAAGAGTTTGTTAAGATGATGGAAAGGAGAGATGCAATCTTCACCATCCAGTACAACGTCCGTTCATTCATGAATGTAAAGAACTGGCCATGGCTTAAACTCTACTTCAAGATCAAGCCTCTTCTGAAGAGCGCTGAGACTGAAAAGGAGCTGCAGGAGATGAAAGGAAACTATGAGAAGATGAAAACAGACCTGGCTGCTGCTTTGGCCAAGAAGAAGGAACTGGAGGAGAAAATGGTTTCTCTGCTGCAGGAAAAGAATGACCTGCAGCTTCAAGTAGCTGCTGAAACAGAGAATCTCTCCGATGCTGAGGAAAGGTGTGAGGGACTCATTAAGAGTAAAATCCAGCTGGAGGCCAAACTCAAAGAGACCACTGAGAGACTGGAGGATGAAGAGGAAATCAATGCTGAGCTGACTGCTAAGAAGAGGAAGCTGGAGGATGAATGTTCTGAACTGAAGAAGGACATTGATGACTTGGAGCTCACCCTGGCTAAAGTGGAGAAGGAGAAACATGCCACTGAAAACAAGGTGAAAAACCTGACTGAGGAAATGGCTTCTCAAGATGAGTCCATTGCAAAGCTGACCAAGGAGAAGAAAGCCCTCCAAGAGGCCCATCAACAAACACTGGATGATCTCCAGGCAGAGGAAGACAAAGTCAACACTCTGACCAAAGCCAAGACCAAGCTGGAACAGCAAGTGGATGATCTTGAAGGTTCATTGGAGCAAGAGAAGAAGATCCGCATGGATCTTGAGAGAGCCAAGAGGAAGCTGGAAGGAGATCTGAAACTAGCCCAGGAATCCATAATGGATCTGGAAAATGACAAGCAGCAGTCTGATGAGAAGATCAAGAAAAAGGACTTTGAAATCAGTCAGCTCCTCAGCAAGATTGAAGATGAACAATCCCTTGGTGCTCAGCTTCAGAAAAAGATCAAGGAACTCCAGGCTCGTATTGAGGAGCTGGAAGAGGAAATTGAGGCTGAGAGAGCTGCCCGGGCCAAAGTAGAGAAGCAGAGAGCTGATCTCTCCAGGGAGCTTGAGGAGATCAGTGAGAGGCTTGAAGAAGCTGGAGGGGCAACAGCTGCTCAGATTGAGATGAACAAGAAGCGGGAAGCTGAGTTCCAGAAGCTGAGGAGAGATCTTGAGGAGTCCACACTGCAGCATGAAGCTACTGCAGCAGCTCTCCGCAAGAAGCAGGCCGACAGCGTTGCAGAGCTGGGAGAACAGATCGACAACCTGCAGCGTGTCAAGCAGAAGCTGGAGAAGGAGAAGAGCGAGTACAAGATGGAGATTGATGATCTCAGCAGCAACATGGAGGCAGTTGCTAAATCAAAAGGTAACCTGGAGAAGATGTGCAGAACTCTTGAGGACCAGCTGAGTGAGCTCAAGGCCAAAAATGATGAAAATGTTCGCCAACTGAATGACATCAATGTACAAAAGGCAAGACTTCAAACGGAAAATGGTGAATTTGCTCGCCAGCTTGAGGAAAAAGAAGCTCTAGTTTCTCAGCTGACCAGAGGCAAACAGGCCTTCACTCAGCAGATTGAGGAGCTGAAGAGACATGTGGAGGAGGAAGTGAAGGCCAAGAACGCTCTGGCTCATGCTGTCCAGTCAGCCCGTCATGACTGTGATCTGCTCAGAGAGCAGTTTGAGGAGGAGCAGGAGGCCAAGGCTGAGCTGCAGAGAGGAATGTCCAAGGCCAACAGTGAGGTGGCTCAGTGGAGAACCAAATATGAAACTGATGCCATCCAGCGCACTGAGGAACTGGAGGAGTCCAAGAAAAAGCTTGCCCAGCGCCTGCAGGAGGCTGAGGAGTCCATTGAGGCTGTGAACTCCAAGTGTGCCTCTCTGGAGAAGACCAAGCAGAGGCTGCAGGGTGAAGTGGAGGACCTCATGATTGATGTGGAGAGAGCTAATGCTCTTGCTGCAAACCTGGACAAGAAACAGAGAAACTTTGACAAGGTCCTGGCAGAATGGAAGCAGAAGTATGAGGAGAGCCAGGCAGAGCTTGAAGGAGCTCAAAAAGAGGCTCGCTCTCTCAGCACAGAACTGTTCAAGATGAAGAACTCCTACGAGGAGGCCCTGGATCAGCTGGAGACCATGAAGAGGGAGAACAAGAACCTGCAGCAGGAGATCTCAGACTTGACTGAACAGATTGGTGAGAATGGAAAAAGCATCCATGAGTTGGAGAAAGCCAAAAAGACTGTTGAAAATGAAAAGATTGAACTTCAGACAGCTCTTGAGGAGGCAGAGGGCACTCTAGAACATGAGGAATCCAAGATTCTTAGAGTACAGCTTGAGCTCAACCAGGTCAAAGGTGAGATTGACAGGAAGCTGGCAGAGAAGGACGAGGAGATGGAACAGATCAAGAGGAACAGCCAGAGGGTGATTGAATCCATGCAGAGCACTCTTGATGCTGAGGTCAGGAGCAGAAATGATGCCCTGAGAGTCAAGAAGAAGATGGAGGGAGACCTGAATGAGATGGAGATTCAGCTCAGCCATGCCAACAGGCAGGCTGCTGAGGCCCAGAAACAACTCAGGAATGTTCAGGGACAACTCAAGGATGCCCAACTGCACCTTGATGACGCTCTGAGAGGACAAGAAGACATGAAAGAACAGGTTGCCATGGTGGAGCGTAGGAATGGTCTGATGTTGGCTGAGATTGAGGAGTTGAGAGCCGCTCTGGAACAGACAGAGAGAGGACGTAAAGTGGCTGAACAGGAGCTGGTGGATGCCAGTGAACGTGTTGCATTGCTTCACTCCCAGAACACCAGTCTTCTGAACACCAAGAAGAAGCTGGAGGCTGACTTTGTTCAGGTTCAGGGTGAAGTGGATGATGCTGTTCAAGAAGCAAGAAATGCAGAGGAGAAAGCCAAGAAGGCCATTACTGATGCTGCCATGATGGCTGAGGAGCTAAAGAAGGAGCAGGACACCAGCTCTCATCTGGAGAGGATGAAGAAGAACCTGGAGGTCACAGTGAAGGACCTGCAGCACCGTCTGGATGAAGCTGAGAACCTGGCTATGAAGGGTGGAAAGAAGCAGCTCCAGAAACTGGAGTCCAGGGTGCGTGAATTAGAGGCTGAAGTTGAAACTGAGCAAAGACGTGGAGCTGATGCGGTTAAAGGCGTTCGCAAATATGAAAGAAGAGTGAAGGAGCTCACATACCAGACTGAGGAGGACAAGAAAAATGTAAGCAGACTCCAGGATCTGGTGGATAAGTTACAGCTCAAAGTAAAGGCCTATAAGAGACAGGCTGAAGAGGCTGAGGAGCAGGCCAACACCCATATGGCCAGACTTAGAAAAGTCCAGCATGAACTTGAGGAAGCTCAGGAACGTGCCGACATCGCAGAGACCCAAGTCAACAAGCTGAGAGCCAAAAGTCGTGATATTGGAAAGGGTACAGACTCAGCTGAGTAA

>Myosinhc (Transcript: ENSORLT00000002485.2): Translation with Smart protein domains annotated (Myosin-N domain, MYSc domain, IQ domain, Myosin-tail-1 domain)

MSTDAEMEAYGPAAIYLRKPEKERIEAQAAPFDAKTAYFVAEPEEMYLKGKLIKREGGKATVETTLTVKEEDIHPMNPPKFDKIEDMAMMTHLNEPAVLYNLKERFASWMIYTYSGLFCVVVNPYKWLPVYDAQVVNAYRGKKRIEAPPHIFSISDNAYQFMLTDRENQSILITGESGAGKTVNTKRVIQYFATIAVAGGKKSEGSSGKMQGSLEDQIIAANPLLEAYGNAKTVRNDNSSRFGKFIRIHFGSTGKLASADIETYLLEKSRVTFQLSAERSYHIFYQLMTGHKPELLEALLITTNPYDYPMISQGEITVKSINDVEEFIATDTAIDILGFSAEEKMGIYKLTGAVMHHGNMKFKQKQREEQAEPDGTEVADKIAYLMGLNSADMLKALCYPRVKVGNEMVTKGQTVPQVNNAVSALCKSVYEKMFLWMVIRINEMLDTKQPRQFFIGVLDIAGFEIFDYNSLEQLCINFTNEKLQQFFNHHMFVLEQEEYKKEGIEWEFIDFGMDLAACIELIEKPMGIFSILEEECMFPKATDTSFKNKLHDQHLGKTKAFEKPKPAKGKAEAHFALVHYAGTVDYNITGWLDKNKDPLNDSVVQLYQKSSNKLLCYLYAAHGGGKKKGGSFQTVSALFRENLGKLMTNLRSTHPHFVRCLIPNESKTPGLMENFLVIHQLRCNGVLEGIRICRKGFPSRILYGDFKQRYKVLNASVIPEGQFIDNKKASEKLLGSIDVDHSQYKFGHTKVFFKAGLLGTLEEMRDEKLAELVTMTQALCRGFLMRKEFVKMMERRDAIFTIQYNVRSFMNVKNWPWLKLYFKIKPLLKSAETEKELQEMKGNYEKMKTDLAAALAKKKELEEKMVSLLQEKNDLQLQVAAETENLSDAEERCEGLIKSKIQLEAKLKETTERLEDEEEINAELTAKKRKLEDECSELKKDIDDLELTLAKVEKEKHATENKVKNLTEEMASQDESIAKLTKEKKALQEAHQQTLDDLQAEEDKVNTLTKAKTKLEQQVDDLEGSLEQEKKIRMDLERAKRKLEGDLKLAQESIMDLENDKQQSDEKIKKKDFEISQLLSKIEDEQSLGAQLQKKIKELQARIEELEEEIEAERAARAKVEKQRADLSRELEEISERLEEAGGATAAQIEMNKKREAEFQKLRRDLEESTLQHEATAAALRKKQADSVAELGEQIDNLQRVKQKLEKEKSEYKMEIDDLSSNMEAVAKSKGNLEKMCRTLEDQLSELKAKNDENVRQLNDINVQKARLQTENGEFARQLEEKEALVSQLTRGKQAFTQQIEELKRHVEEEVKAKNALAHAVQSARHDCDLLREQFEEEQEAKAELQRGMSKANSEVAQWRTKYETDAIQRTEELEESKKKLAQRLQEAEESIEAVNSKCASLEKTKQRLQGEVEDLMIDVERANALAANLDKKQRNFDKVLAEWKQKYEESQAELEGAQKEARSLSTELFKMKNSYEEALDQLETMKRENKNLQQEISDLTEQIGENGKSIHELEKAKKTVENEKIELQTALEEAEGTLEHEESKILRVQLELNQVKGEIDRKLAEKDEEMEQIKRNSQRVIESMQSTLDAEVRSRNDALRVKKKMEGDLNEMEIQLSHANRQAAEAQKQLRNVQGQLKDAQLHLDDALRGQEDMKEQVAMVERRNGLMLAEIEELRAALEQTERGRKVAEQELVDASERVALLHSQNTSLLNTKKKLEADFVQVQGEVDDAVQEARNAEEKAKKAITDAAMMAEELKKEQDTSSHLERMKKNLEVTVKDLQHRLDEAENLAMKGGKKQLQKLESRVRELEAEVETEQRRGADAVKGVRKYERRVKELTYQTEEDKKNVSRLQDLVDKLQLKVKAYKRQAEEAEEQANTHMARLRKVQHELEEAQERADIAETQVNKLRAKSRDIGKGTDSAE

>pAP27 insert: Linker-3xFlag- mNeonGreen-HAtag-Linker

TCTAGCGGACCTTCAGGTAGTTCGAGCGACTACAAGGACCACGACGGGGATTATAAAGATCATGATATAGATTATAAAGATGATGATGATAAAGTCTCCAAAGGGGAGGAAGACAACATGGCGTCCCTCCCAGCGACGCACGAACTCCATATCTTTGGGTCCATAAACGGTGTCGATTTCGACATGGTAGGACAAGGGACCGGAAATCCAAATGATGGATATGAGGAGCTAAACCTCAAGAGTACCAAGGGGGACCTCCAATTTTCCCCATGGATACTCGTCCCCCATATCGGGTACGGATTCCATCAATACCTACCATATCCAGATGGGATGTCCCCATTTCAAGCGGCCATGGTAGACGGAAGTGGATACCAAGTACATCGTACGATGCAATTTGAGGACGGGGCCTCCCTAACCGTCAACTATCGTTACACGTACGAGGGAAGTCACATCAAAGGAGAGGCGCAAGTCAAAGGAACTGGATTTCCAGCCGATGGACCAGTCATGACGAATTCCCTAACCGCCGCCGATTGGTGTCGTAGTAAAAAGACGTACCCAAATGACAAGACCATAATCTCCACGTTCAAGTGGTCCTATACCACCGGGAACGGAAAGCGGTACCGTAGTACGGCCCGGACGACGTACACGTTCGCCAAGCCCATGGCCGCTAATTACCTGAAGAATCAGCCAATGTATGTCTTCCGTAAAACCGAGCTAAAGCACTCCAAAACCGAGCTAAACTTCAAGGAATGGCAAAAGGCGTTCACGGACGTAATGGGAATGGACGAACTCTACAAGTACCCATATGATGTCCCGGATTACGCTAGCTCTGGTCCAAGTGGATCAAGCTCG

>*mNeonGreen-HAtag-Linker-myosinhc* (Transcript: ENSORLT00000002485.2): with mNeonGreen-HAtag-Linker, coding exons are in grey, double underlined corresponds to PCR primer sequences, PAM in bold

CAAACATGCTTAGTAGGCAATATAAGCACTGTAAATATCAATGTCACAGGATTAGAGACAACATGACTAGAATACATTACCAAATTGAGCAGGGCACACCTACCACACAGTCAGTCCTCCCTCTATAAAAGAGAGCACTTAATGCTCTGGGAATAACTGGTGGGCTTCCAACTCAGGTGAGTGCAAAAATGTATTAAGCCTTATTTTGCAAATTTCTGTTGTTCAAATGCTATTTGGAATATAATTGTTCCCAAAGGAAATCTATAAACAAATGGGATGCATTTAGATTTGTTTTAAGTCATTGTATTGTATTAAGATGCTTGTGCTTTACAATTTAATAGTGGAAACAACATAACACCGTAAGATAATCTGCTGAGAGCCAAACAGGTAGACATTTTTTTTCCAAATTCTTTACCATGTTTCTTGTTTTGAGCAAGTGAACAAAAAATAAATAAATCAATGATTGTCACCCCTCATTTTTAGACATCTCAAGTGCTA**CCA**TGGTCTCCAAAGGGGAGGAAGACAACATGGCGTCCCTCCCAGCGACGCACGAACTCCATATCTTTGGGTCCATAAACGGTGTCGATTTCGACATGGTAGGACAAGGGACCGGAAATCCAAATGATGGATATGAGGAGCTAAACCTCAAGAGTACCAAGGGGGACCTCCAATTTTCCCCATGGATACTCGTCCCCCATATCGGGTACGGATTCCATCAATACCTACCATATCCAGATGGGATGTCCCCATTTCAAGCGGCCATGGTAGACGGAAGTGGATACCAAGTACATCGTACGATGCAATTTGAGGACGGGGCCTCCCTAACCGTCAACTATCGTTACACGTACGAGGGAAGTCACATCAAAGGAGAGGCGCAAGTCAAAGGAACTGGATTTCCAGCCGATGGACCAGTCATGACGAATTCCCTAACCGCCGCCGATTGGTGTCGTAGTAAAAAGACGTACCCAAATGACAAGACCATAATCTCCACGTTCAAGTGGTCCTATACCACCGGGAACGGAAAGCGGTACCGTAGTACGGCCCGGACGACGTACACGTTCGCCAAGCCCATGGCCGCTAATTACCTGAAGAATCAGCCAATGTATGTCTTCCGTAAAACCGAGCTAAAGCACTCCAAAACCGAGCTAAACTTCAAGGAATGGCAAAAGGCGTTCACGGACGTAATGGGAATGGACGAACTCTACAAGTACCCATATGATGTCCCGGATTACGCTAGCTCTGGTCCAAGTGGATCAAGCTCGAGCACTGACGCAGAGATGGAGGCCTATGGCCCTGCGGCCATCTACCTCCGGAAACCAGAGAAGGAGAGGATTGAGGCTCAGGCAGCTCCTTTTGATGCCAAAACGGCCTACTTTGTGGCAGAGCCAGAGGAGATGTATCTGAAGGGAAAACTTATCAAAAGGGAGGGTGGCAAAGCCACTGTTGAGACAGTAACAGGAAAGGTTGGTAAACTAAGGCTTTTGTTTACTCTTACATTTCACTTACCAAGTAAAATGTAATCAACTCTATGTCATTTAAGTAAACCATAAATCATGTGAAACACTGGTTAAAAATGTTCTTATTTATTCTAACTCTTGTTTAGACTTTAACTGTGAAAGAGGAGGACATCCATCCAATGAACCCTCCCAAGTTTGATAAAATTGAGGACATGGCCATGATGACCCACCTTAATGAACCCGCTGTGCTGTACAACCTCAAAGAACGTTTTGCATCATGGATGATCTATGTATGTAAATGACTTAGACAAGCACAGAGAAAAAATGTTTTATTTTATATTGGTGTAACGTAACGTTTCAAAAGTTTACTATTTGGTTTAGCAATAACTCTACTGTCTTTACAGACTTACTCTGGGCTGTTTTGTGTTGTCGTGAACCCATACAAGTGGCTTCCGGTGTACGATGCTCAGGTTGTCAATGCCTACAGAGGCAAGAAGAGAATTGAGGCTCCCCCTCATATCTTTTCCATCTCTGACAATGCCTATCAGTTCATGCTCACTGGTAAGATAGTTGCACAACAGAGAAAGCAGCACACACAAATAAAACTCCACAGGTGTGGTTCACTGTGCGCATGCTTGCATTTCAGATCGTGAGAATCAGTCTATCCTTATCACGTGAGTATAAGGCAACATGCCAAGAGTCCCTATGAAATTTTAGGATTAATGTGCCACATAAAGGCTGTGTCTTACATTCTCAGTGGAGAATCTGGTGCAGGAAAGACTGTCAACACCAAGCGTGTCATCCAGTACTTTGCAACAATTGCAGTGGCTGGAGGAAAGAAGTCTGAGGGAAGCTCAGGAAAAATGCAGGTACAGAAGAATGCAAAACTTCTCACAATAGTAGGTTGTTGTTTAGTTAAAACACATTAGTTTGTTCTTTCTGCAGGGTTCACTGGAAGATCAAATCATTGCAGCCAACCCTCTGCTGGAGGCCTATGGTAATGCCAAGACTGTGAGGAATGATAACTCTTCTCGCTTTGTGAGTTTAAAAAAAGTCCTCAAATATTGTTAATGTTCTTATTACTTAGAATTTGTCTGACATTGTCTTGAAAATTTTCAGGGTAAATTTATCAGAATCCACTTTGGTTCTACTGGTAAACTTGCTTCAGCTGATATTGAAACATGTAAGTTAGACAGCATTTCTATAATTGTAACTCCTTGGCCTGCATCGGTGCAGGACTAACATTAACAACATGCAATAAATTTCAGATCTGCTGGAGAAGTCTCGTGTCACCTTCCAGTTGTCTGCTGAGAGGAGCTACCATATCTTTTACCAGCTGATGACGGGCCACAAACCTGAGCTCCTTGGTATGATACAAGACAAATAATTACACTTAGGATGGAATAGGTAAAAAAAATCCTCTTTACTTGTGTTATTTTCAAGGTACATAAAGAGAAAACTACTTTTCCTTCTAGAGCAAATCTGTTACCAAAAATGTACATGCAAAAGAGAATGTTCCCATTTCTGATATTCCATTTTTTCCCCTTTAGAGGCTCTTCTTATCACCACCAACCCGTATGATTATCCAATGATCAGCCAGGGTGAAATCACTGTCAAGAGCATCAATGACGTTGAGGAGTTCATTGCAACTGATGTAAAAGAAATACTTAATACTTGTAAATTATAATTTGCAAAATTGTAATCATTAATAATTGAACTATTTTAATTTTTAGGTCTGTACATATCAATTTATGTTATGGGATTTTTTAAAACAATTGTCTCTGTATGTTTGCAGACTGCCATTGACATATTGGGATTCAGTGCTGAGGAAAAAATGGGCATCTACAAGCTAACTGGTGCTGTGATGCATCATGGTAACATGAAGTTTAAGCAGAAGCAGCGAGAGGAGCAGGCAGAACCTGACGGCACAGAGGGTAAATTAACTGTTAATAATGATAGATCAATAATGACTTAGTCTCAGTTTGTTTTACATTTTATAAGATAAGATAAGATAAGATAAAGATACTTTATTGATCCCAGCTTGGGAAATTCAGTTGTTGCAGCGTCGGCATTATAGGCTCAAGATACAAACATACACTCAATAGACGACAACAGAGAACCAGAACAATCAATCGGAAAGGAACATCAGTGAATTAGAACATTTGCATTGCGTGCCACATCAGGAGAGAAAAACAGACAGACCATAAATGACTTTATCTAGTGCCCATGGATGACAAGTTATAGAGTCCAATGGAGTGAGGTATGAAAGATCTCCTGAAGCGGTTTGTGTGCCAGCGAAGCTGCCTTAGCCTGTTGGAGAAGGAGCTCCTCTGACCATCCAGTACATCCAGTACAGTTTTACTCAACACAGAGGAAAATGCTTTGTTAAATCCAGAATTGTTTTTTCCATCAGTGGCTGATAAAATCGCCTACCTGATGGGCCTAAACTCAGCTGACATGCTGAAAGCTCTGTGCTACCCAAGAGTCAAGGTTGGAAATGAGATGGTCACCAAAGGTCAGACTGTCCCACAGGTGACTCACTCAAACAGTTTATGGGGATTGTTTACTTAAAAGAAATATTGATTTGAAATAATTTCATTAAAGTGCTAAACTTTTTTTTAGGTTAACAATGCTGTCAGTGCTCTGTGCAAGTCTGTCTATGAGAAAATGTTCTTGTGGATGGTCATCCGCATCAATGAGATGTTGGACACAAAGCAGCCAAGACAGTTCTTCATTGGAGTGCTGGACATTGCTGGATTTGAAATCTTTGATGTGAGTTGCAGTGGGTAACAAATTAGGCTATCAGTCATTTTCCTATATTTTAAGGAGTTAAACCTGTTTTGTTTTCAACCAAATAATCCTTTTTTTTAACCTGCAGTACAACAGCTTGGAGCAGCTCTGCATCAACTTCACCAATGAGAAACTGCAACAGTTTTTCAATCACCACATGTTTGTCCTGGAGCAAGAGGAGTACAAGAAAGAAGGCATTGAATGGGAGTTCATTGACTTTGGCATGGACTTGGCTGCCTGCATTGAGCTTATTGAGAAGGTATACATTCTGTTCAGAAATGTTCCCGTTGTGAGATTAGTAACGTTCTACCTATTTATCCTTGTGTAATTACTTTTTATGATTAGGAAAAAAAAGTTTTTTCATCTTGGTCTGCTATTTTAATGCCAAATTATACTTTTTCCAAGCCAATGGGCATCTTCTCCATCCTTGAAGAGGAGTGCATGTTCCCCAAGGCTACAGACACCTCATTTAAGAACAAACTGCATGATCAGCATCTTGGCAAGACCAAGGCCTTTGAGAAGCCTAAACCTGCAAAAGGAAAAGCTGAAGCTCACTTTGCCCTGGTTCATTATGCTGGAACAGTGGATTACAACATAACTGGCTGGCTGGACAAAAACAAGGACCCACTGAACGACTCAGTGGTGCAGCTCTACCAGAAGTCTTCAAACAAACTGCTGTGCTACCTCTATGCAGCCCATGCAGGAGCTGAAGGTAAGACTTTAACAAAAAGCTGTTAAATGATAGTTTGTTTATATCTAGATAAATGCACATCATTAAAATTCATGTGTAGTATGACTAATTGTGTTATATATATCAAAAACAAAACAAAATTAGAAGCCTCTGGTGGTGCCAAAAAGGGTGGTGGAAAGAAGAAGGGTGGTTCCTTCCAAACTGTGTCAGCACTTTTCAGGGTAAGTTTATTTATTAAGACATATTTAAGATCTGTAGATTTTATTTTTTTACAACTGGCACTGAAAAAAATTATGTGCTTCATTCATCAGGAAAATCTTGGCAAGCTGATGACCAATTTGAGAAGCACTCATCCACATTTTGTCCGCTGCTTGATTCCAAATGAATCTAAAACCCCAGGTCTGTTGTGGTACCTTATAACTACGTCAAAGTCAATATATGATTTGAAAAATTCCCAGAAACAATAAAAAGGGGCTACCCTTTCTTTTACAATTCCTTGTATTTTAAAACTTCATCCATTCTGAATGATTAGGTCTTATGGAGAACTTCTTGGTCATCCACCAGCTGAGGTGTAATGGTGTGCTGGAGGGCATCAGAATCTGCAGAAAGGGTTTCCCCAGCAGAATCCTCTATGGTGACTTCAAGCAGAGGTAAATGGCAGATTTACTGGATGTAATTTAAAAAAAAAATATATATATATATATATATGTGTGTGTGTATATATATAAATATATATATATATATATATATATATATATATATATATATATATATATATATATATATATATGTGTGTGTGTGTGTATATATATATAAATATATAACTTTGCTAAATAATATTAATTAGAAAGGAAAATGTGGATAAATCTACTTTTATCTTACAGATACAAAGTATTGAATGCCAGTGTCATCCCTGAGGGACAGTTCATCGACAACAAGAAAGCTTCAGAGAAGCTGCTTGGTTCCATTGATGTTGACCACAGTCAGTACAAGTTTGGACACACTAAGGTAGGTTTCTATAAAATAGTTATGTAAATTGGCTCAAGTCCCAGCTGGGGAACCTGAAACAGAACACCAATGTGGGACCCTGTGTGGAGATTGCATGTTCTTCCTGTCCATGTTTGGGTTTTCTCCTCCATTCAAAAACGTGCTTCATAGGTTAGCAGATTACTCTGAATTGTCCCTAGGTGTGAATGTGAGTGTGCTTGGGCCTATCCAGGCCACAAGTGACTGGGATAGGCTCCAGCAGCCCTGTGACCCCGAAAGGGACAAAATGGCTTAATGAATGAATGAATGAATGAATGAATGAATGAATGAATGAATGAATGAATGAATGAATGAATGAATGAATGAATGAATGAATGAATGAATGAATGAATGAATGAATTGGCTTTTGGACGAGGTGTAACCTATCATGTCAATATTTCAACACAGGTGTTCTTCAAAGCTGGTCTGCTGGGTACACTTGAGGAGATGAGAGATGAGAAGCTGGCTGAGCTGGTGACCATGACTCAGGCTCTCTGCAGAGGGTTCCTTATGCGGAAAGAGTTTGTTAAGATGATGGAAAGGAGGTAAGACTCCCACATATTAGCTTACACAGGAATCACTGTTCAAATTAAAAGGCTGTTCAGGAAGCATAATGTCAACTTATCTGTGTTGCTCTTTAGAGATGCAATCTTCACCATCCAGTACAACGTCCGTTCATTCATGAATGTAAAGAACTGGCCATGGCTTAAACTCTACTTCAAGATCAAGCCTCTTCTGAAGAGCGCTGAGACTGAAAAGGAGCTGCAGGAGATGAAAGGAAACTATGAGAAGATGAAAACAGACCTGGCTGCTGCTTTGGCCAAGAAGAAGGAACTGGAGGAGAAAATGGTTTCTCTGCTGCAGGAAAAGAATGACCTGCAGCTTCAAGTAGCTGCTGTAAGTAAAAAATATGAAGACATATCATGAACAAAGCTCATAAAATAATGTCTTATATCAATATTTAGGAATAAAAATAATGTGACAAAATATGTTTGATATTAGATGTCAGAAATGTAGAAATGTGTCTAAACTACAGGAAACAGAGAATCTCTCCGATGCTGAGGAAAGGTGTGAGGGACTCATTAAGAGTAAAATCCAGCTGGAGGCCAAACTCAAAGAGACCACTGAGAGACTGGAGGATGAAGAGGAAATCAATGCTGAGCTGACTGCTAAGAAGAGGAAGCTGGAGGATGAATGTTCTGAACTGAAGAAGGACATTGATGACTTGGAGCTCACCCTGGCTAAAGTGGAGAAGGAGAAACATGCCACTGAAAACAAGGTTAATGTCAAACTATTGTACACATTTTATATAAATTCAAATTATAAAATTTGACCTTTTATGGTACTCTAAGGTGAAAAACCTGACTGAGGAAATGGCTTCTCAAGATGAGTCCATTGCAAAGCTGACCAAGGAGAAGAAAGCCCTCCAAGAGGCCCATCAACAAACACTGGATGATCTCCAGGCAGAGGAAGACAAAGTCAACACTCTGACCAAAGCCAAGACCAAGCTGGAACAGCAAGTGGATGATGTAAGAAAAATTAATGTTTTGACAAATTTGACTGAAATAGATGATCTGAAGAAGTTATTTAACAATGAAATATTTTTATCTGACAGCTTGAAGGTTCATTGGAGCAAGAGAAGAAGATCCGCATGGATCTTGAGAGAGCCAAGAGGAAGCTGGAAGGAGATCTGAAACTAGCCCAGGAATCCATAATGGATCTGGAAAATGACAAGCAGCAGTCTGATGAGAAGATCAAGAAGTAAGAAAAAATGTGTTTTGATGCAAAATTAAAGGGTTTTTTGTGGTTTTAAAGTAAACGGGGACATTCCTTTTTGAAATCCAGAAAGGACTTTGAAATCAGTCAGCTCCTCAGCAAGATTGAAGATGAACAATCCCTTGGTGCTCAGCTTCAGAAAAAGATCAAGGAACTCCAGGTACAATTTTTGCAGCCATTATTGGCTAGATTATTGTGGGTTGTTGTAATACACTACTTATATATATTAAGAGTATTGTATATAATACTCTACTTATAATACAAAACTTTAAAAAATTTAATTTAGGCTCGTATTGAGGAGCTGGAAGAGGAAATTGAGGCTGAGAGAGCTGCCCGGGCCAAAGTAGAGAAGCAGAGAGCTGATCTCTCCAGGGAGCTTGAGGAGATCAGTGAGAGGCTTGAAGAAGCTGGAGGGGCAACAGCTGCTCAGATTGAGATGAACAAGAAGCGGGAAGCTGAGTTCCAGAAGCTGAGGAGAGATCTTGAGGAGTCCACACTGCAGCATGAAGCTACTGCAGCAGCTCTCCGCAAGAAGCAGGCCGACAGCGTTGCAGAGCTGGGAGAACAGATCGACAACCTGCAGCGTGTCAAGCAGAAGCTGGAGAAGGAGAAGAGCGAGTACAAGATGGAGATTGATGATCTCAGCAGCAACATGGAGGCAGTTGCTAAATCAAAAGTGAGAATTGTCATTGATTTTCTTTTGAGAACTCTGATAAATATAACCTTATAACGTTTGCTTTTATTTTTGTCTATTTCTTTTAAGGGTAACCTGGAGAAGATGTGCAGAACTCTTGAGGACCAGCTGAGTGAGCTCAAGGCCAAAAATGATGAAAATGTTCGCCAACTGAATGACATCAATGTACAAAAGGCAAGACTTCAAACGGAAAATGGTGAGTAACTTGAAACAAAAAGGTATTTTTTAAGATTACAATTAAAATTAAGTAAACTGTTTTTGCTTGTTATTATCAGGTGAATTTGCTCGCCAGCTTGAGGAAAAAGAAGCTCTAGTTTCTCAGCTGACCAGAGGCAAACAGGCCTTCACTCAGCAGATTGAGGAGCTGAAGAGACATGTGGAGGAGGAAGTGAAGGCATGAAAACCTTATGCATTCTATTAATCTCAAGCTTCTTGGAGGCTGTAAAAATTCTGGGCCCCCTAAATAAATTTTAATAAATCATTTTAAAACAATCAAGTCACTGAAGAGTTCTGTGATTTCTCCAGGCCAAGAACGCTCTGGCTCATGCTGTCCAGTCAGCCCGTCATGACTGTGATCTGCTCAGAGAGCAGTTTGAGGAGGAGCAGGAGGCCAAGGCTGAGCTGCAGAGAGGAATGTCCAAGGCCAACAGTGAGGTGGCTCAGTGGAGAACCAAATATGAAACTGATGCCATCCAGCGCACTGAGGAACTGGAGGAGTCCAAGTTAGTCAAAGCAGTTCATCATTTCATCAAACTATTCTGTCTCTCACTTCTTTTAAAACATTCACTCCATCCATTTGCTGCAGGAAAAAGCTTGCCCAGCGCCTGCAGGAGGCTGAGGAGTCCATTGAGGCTGTGAACTCCAAGTGTGCCTCTCTGGAGAAGACCAAGCAGAGGCTGCAGGGTGAAGTGGAGGACCTCATGATTGATGTGGAGAGAGCTAATGCTCTTGCTGCAAACCTGGACAAGAAACAGAGAAACTTTGACAAGGTACCATGTTCATCTGACAGTTAAAAAAAGTAAAACATGTTGGGAAGATACACTTACTTTAGTAACAAACCTGCAACCAATGGTTAACCTCTAGGTCCTGGCAGAATGGAAGCAGAAGTATGAGGAGAGCCAGGCAGAGCTTGAAGGAGCTCAAAAAGAGGCTCGCTCTCTCAGCACAGAACTGTTCAAGATGAAGAACTCCTACGAGGAGGCCCTGGATCAGCTGGAGACCATGAAGAGGGAGAACAAGAACCTGCAGCGTATGTCAACAATCTATATTGATAACAAAGAATTATTTATAACCAATTGGAAAGATACAAATTTTGCTAACTTTTCTTGAATTAACATGACAGAGGAGATCTCAGACTTGACTGAACAGATTGGTGAGAATGGAAAAAGCATCCATGAGTTGGAGAAAGCCAAAAAGACTGTTGAAAATGAAAAGATTGAACTTCAGACAGCTCTTGAGGAGGCAGAGGTAAAATTTTGTAAACAAGATATTGTGTATTAAGTTTACACTTCACAATTTTTTTCACAGTTGTCTTGTTAATTAACTTTGGCCTTTTCCTTCAGGGCACTCTAGAACATGAGGAATCCAAGATTCTTAGAGTACAGCTTGAGCTCAACCAGGTCAAAGGTGAGATTGACAGGAAGCTGGCAGAGAAGGACGAGGAGATGGAACAGATCAAGAGGAACAGCCAGAGGGTGATTGAATCCATGCAGAGCACTCTTGATGCTGAGGTCAGGAGCAGAAATGATGCCCTGAGAGTCAAGAAGAAGATGGAGGGAGACCTGAATGAGATGGAGATTCAGCTCAGCCATGCCAACAGGCAGGCTGCTGAGGCCCAGAAACAACTCAGGAATGTTCAGGGACAACTCAAGGTGAGATTTATCTTCAATTAAAGCTACTAACTTATGAACATCTTTACACAACTTTATGAAAAAAAAATTGTTTCAAAGTAGCAGACTTCTAAAAGAACTTCTAGAAAGTTTTATCAGCGTCACGCATGCTCTTATTGTTGTTGTCAGGATGCCCAACTGCACCTTGATGACGCTCTGAGAGGACAAGAAGACATGAAAGAACAGGTTGCCATGGTGGAGCGTAGGAATGGTCTGATGTTGGCTGAGATTGAGGAGTTGAGAGCCGCTCTGGAACAGACAGAGAGAGGACGTAAAGTGGCTGAACAGGAGCTGGTGGATGCCAGTGAACGTGTTGCATTGCTTCACTCCCAGGTTGATATTTAACAAAAACTGAAAAACTGTGCACAACTCAAGATTTGAGAAACGTTCACAGGCAATCAGGCAATAATTACATTTATTTTCAGAACACCAGTCTTCTGAACACCAAGAAGAAGCTGGAGGCTGACTTTGTTCAGGTTCAGGGTGAAGTGGATGATGCTGTTCAAGAAGCAAGAAATGCAGAGGAGAAAGCCAAGAAGGCCATTACTGATGTAAGTTTATTCTTGGTACATTTCAAACATTGGATGCTCGTGTTTACCACTGATTTCTTTTCAGTTTAAATTAAATCAGACACCTGTTATCCCTTTCAGGCTGCCATGATGGCTGAGGAGCTAAAGAAGGAGCAGGACACCAGCTCTCATCTGGAGAGGATGAAGAAGAACCTGGAGGTCACAGTGAAGGACCTGCAGCACCGTCTGGATGAAGCTGAGAACCTGGCTATGAAGGGTGGAAAGAAGCAGCTCCAGAAACTGGAGTCCAGGGTAGATACATAGTGTCCCTAATTTTGCAATATTTATATTGTGTGGGTTTGTTCTGTCTGTAGTGTACTAACTCTGCTCTTTAACATCCTATTATAGGTGCGTGAATTAGAGGCTGAAGTTGAAACTGAGCAAAGACGTGGAGCTGATGCGGTTAAAGGCGTTCGCAAATATGAAAGAAGAGTGAAGGAGCTCACATACCAGGCAATTCAAACTCTTTTTACAGATTTCCGTCTTAATTTTGAATAGATTTAATTTTAAGTCAAAACTTTTTAATCTGTGTAGACTGAGGAGGACAAGAAAAATGTAAGCAGACTCCAGGATCTGGTGGATAAGTTACAGCTCAAAGTAAAGGCCTATAAGAGACAGGCTGAAGAGGCTGTAAGTAACATTCTATTAGGATTTGGTTAGGAAAATAAAAATGTAACTAGTTTAACAACCACATTATTTCACTCTTTTCATTTTATAGGAGGAGCAGGCCAACACCCATATGGCCAGACTTAGAAAAGTCCAGCATGAACTTGAGGAAGCTCAGGAACGTGCCGACATCGCAGAGACCCAAGTCAACAAGCTGAGAGCCAAAAGTCGTGATATTGGAAAGGTAACTTTTCACTCTGTGATATTTTCATATATCTAATTAATGATAAGCACATGTGTAATTTATTACAGTTCAAATGTAAACAAAAAGAATTGTGTTACAAAGAATTCCCAAAATTATATTGCGAACAACAGGTTTCTCTCACAACCTCTTACCCAACAAAATGTCTTCTTCTTTCAGGGTACAGACTCAGCTGAGTAACAGTCAAGATCCCCTGGGACAGAGACAGATTCATATAATATGAACCCCCTCTGTTCATCTTTAGCTTATAAATAAATGAACTATACTTGAAACAAATTCCGTGTATTGTTCATTTACTTCTTTATTGTGGTCAGAGTCAATAATAGCTGAAGATGTCTTCAGGCAAGATGCTGTATATTCTTTGAGTGCTTGTCAGTCTATCACAAATCACCTCATCTCATAAAACTGTATGAGAGTTAATATAAGACAAAGATAAATAATCAGTCATATCATTGCTGTAATTCAAGTTGTAACATGTATTAATGCTCTTTCATTTGAGGCTGCAGCGTGGGGAGGCTGGTTACAAATTTAAATCAAACATTCAAACGAAATTAAGACCTTTTCTGTTTGGTATTTTGCTTTTATTATTATACATCTTCATTATCCTACCTTAACACGAGTCACAAGAGCACTGGGCTTACTTACACATGATGACAACAGTAAAACAGATAAACACAAAA

>Primer F (5’to3’): AP254

5'Biotin-CCCCTCATTTTTAGACATCTCAAGTGCTACCATGGTCTCCAAAGGGGAGGAAG

>Primer R (5’to3’): AP255

5'Biotin-GCAGGGCCATAGGCCTCCATCTCTGCGTCAGTGCTCGAGCTTGATCCACTTGGAC
